# From functional foods to immunotherapeutic agents: mechanistic insights into medicinal mushroom bioactives in chronic inflammation management

**DOI:** 10.3389/fnut.2025.1725297

**Published:** 2025-12-08

**Authors:** Ma Xiaoying, Zhang Peng, Wang Hong, Gong Na, Xiao Jun, Zhao Ying, Chen Xun, Liu Guoli

**Affiliations:** The Institute of Edible Fungi, Liaoning Academy of Agricultural Sciences, Shenyang, China

**Keywords:** medicinal fungi, immunomodulation, chronic inflammation, β-glucans, precision medicine

## Abstract

**Background:**

Chronic inflammation underlies numerous complex diseases, yet current therapeutic strategies show limited efficacy and safety profiles. Despite extensive preclinical evidence, the mechanistic understanding and clinical translation of medicinal mushroom bioactives remain inadequately characterized.

**Objective:**

This review systematically evaluates the immunoregulatory mechanisms of mushroom-derived bioactive compounds and establishes a comprehensive framework for their therapeutic application in chronic inflammatory diseases.

**Methods:**

We analyzed mechanistic evidence for four major compound classes: polysaccharides (β-glucans), triterpenoids, phenolic compounds, and bioactive peptides, examining their effects on immune cell populations and signaling pathways.

**Results:**

These bioactives demonstrate multi-target anti-inflammatory activity by modulating key cellular mediators (macrophages, regulatory T cells, natural killer cells) and critical signaling cascades (NF-κB, MAPK, NLRP3 inflammasome, Nrf2/HO-1). Novel therapeutic targets including gasdermin-mediated pyroptosis provide additional intervention opportunities. However, clinical translation faces significant challenges: poor bioavailability, lack of standardization, and undefined dose–response relationships.

**Conclusion:**

Advanced delivery systems (nanoformulations, structural optimization) and precision nutrition approaches through personalized immune profiling offer promising solutions to overcome translational barriers. This analysis provides evidence-based rationale for advancing medicinal mushrooms from traditional functional foods to standardized immunotherapeutic agents for chronic inflammation management.

## 1 Introduction

Chronic inflammation constitutes a fundamental pathophysiological mechanism underlying diverse diseases, including cardiovascular disorders, metabolic syndrome, neurodegenerative conditions, and autoimmune diseases (1, 2). Current therapeutic approaches predominantly rely on synthetic anti-inflammatory agents, which often present limited efficacy profiles and significant adverse effects, particularly with long-term use (3). This therapeutic gap has intensified interest in natural immunomodulatory agents that can provide sustainable inflammation management with improved safety profiles.

Medicinal mushrooms have emerged as promising candidates for chronic inflammation management, with over 2,000 species demonstrating documented bioactive properties (4, 5). Recent comprehensive reviews have established the fundamental immunomodulatory potential of mushroom-derived compounds (6, 7), yet critical gaps remain in understanding their precise mechanistic actions and translational applicability. Unlike previous reviews that primarily focused on individual compound classes or single species, the mechanistic understanding of multi-compound synergy and species-specific efficacy variations remains inadequately characterized.

The principal bioactive constituents responsible for anti-inflammatory activities encompass structurally diverse chemical classes: polysaccharides (particularly β-glucans), triterpenoids, phenolic compounds, and bioactive peptides (8). However, significant knowledge gaps persist regarding their comparative therapeutic potency, optimal concentration ranges, and species-specific bioactivity profiles. For instance, *β*-glucan preparations from *Ganoderma lucidum* demonstrate IC₅₀ values of 15–50 μg/mL for inflammatory cytokine inhibition, while *Cordyceps militaris* polysaccharides show efficacy at 25–75 μg/mL, indicating substantial interspecies variability that requires systematic evaluation (9).

Current mechanistic understanding reveals that mushroom bioactives modulate key inflammatory signaling pathways, including nuclear factor-κB (NF-κB), mitogen-activated protein kinase (MAPK), NOD-like receptor protein 3 (NLRP3) inflammasome, and nuclear factor erythroid 2-related factor 2/heme oxygenase-1 (Nrf2/HO-1) pathways (10). However, contradictory findings exist regarding pathway selectivity and compound-specific targeting preferences. For example, ganoderic acids from *G. lucidum* demonstrate preferential NF-κB inhibition with minimal MAPK interference, while cordycepin from *C. militaris* shows broad-spectrum pathway modulation, suggesting distinct mechanistic profiles that warrant comparative analysis (11).

Critical translational challenges significantly limit clinical implementation despite promising preclinical evidence. Poor aqueous solubility, low gastrointestinal absorption (bioavailability often <5%), and inconsistent standardization across mushroom-derived products present substantial barriers to therapeutic application (9, 12). Furthermore, significant pharmacokinetic variations exist between species and extraction methods, with Shiitake (*Lentinula edodes*) lentinan showing 12-h half-life compared to 4–6 h for *G. lucidum* polysaccharides, indicating the need for species-specific pharmacokinetic optimization (10, 11).

The distinction between preclinical efficacy and clinical translatability remains poorly defined. While numerous *in vitro* and animal studies demonstrate anti-inflammatory effects, human clinical trials are limited and show inconsistent outcomes (12, 13). This disparity highlights the urgent need for standardized evaluation frameworks that can bridge preclinical promise with clinical validation.

Recent advances in nanotechnology-based delivery systems and precision medicine approaches offer promising solutions to overcome these translational barriers. Nanoformulation strategies have demonstrated up to 10-fold bioavailability enhancement, while personalized immune profiling approaches enable patient-specific therapeutic optimization (1, 14). However, the integration of these innovative approaches with mushroom bioactives requires systematic investigation and validation.

**Figure 1:**
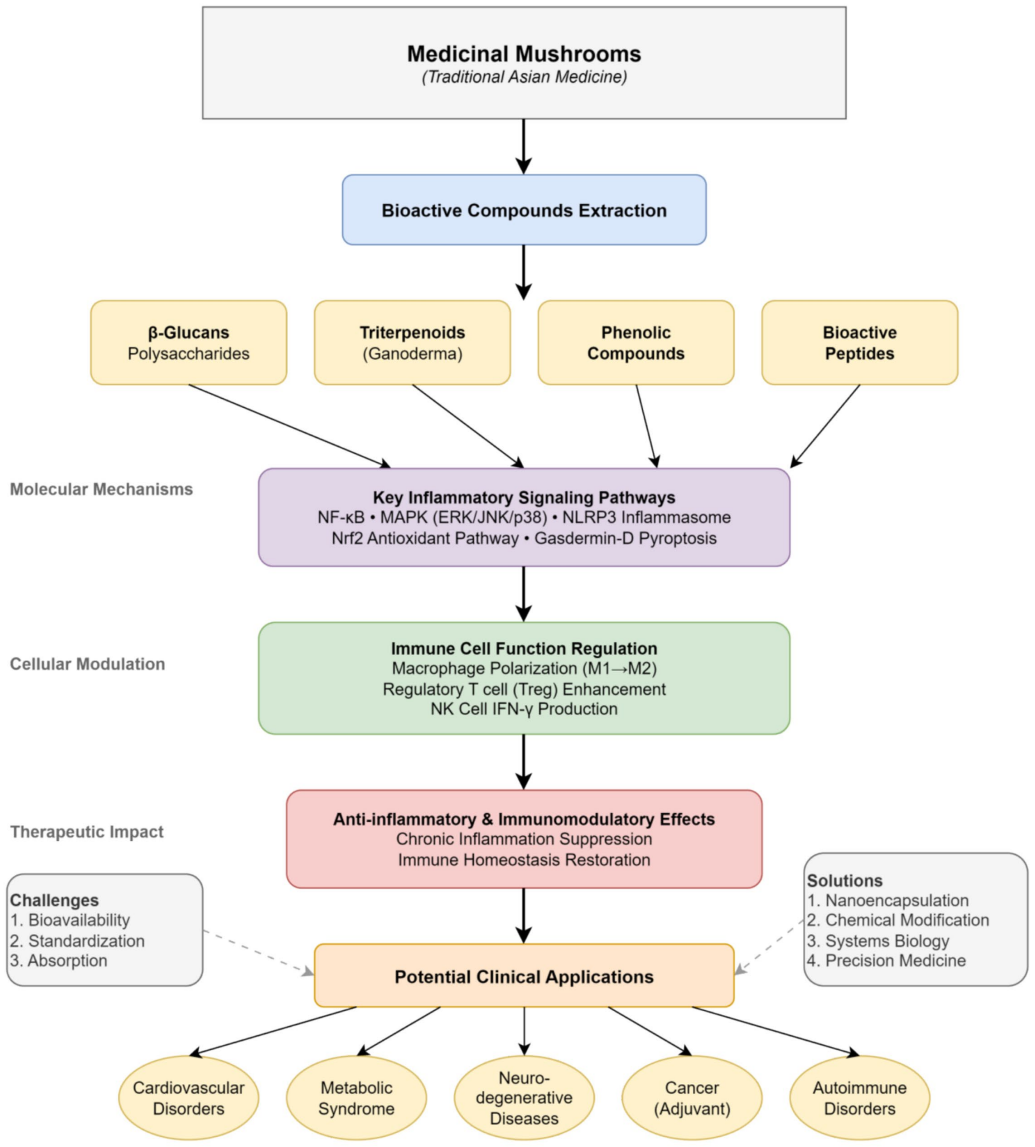
Comprehensive framework of immunoregulation by bioactive compounds from medicinal mushrooms in chronic inflammation.

However, despite extensive documentation of the pharmacological potential of mushroom-derived bioactives, a systematic integration of their molecular mechanisms, synergistic interactions, and translational implications remains limited (4). This review advances beyond the existing literature through four key contributions: First, we construct an integrated mechanistic framework that systematically connects immune regulation at the cellular level—such as macrophage polarization and regulatory T cell induction—with molecular signaling networks, including NF-κB, MAPK, the NLRP3 inflammasome, and the Nrf2/HO-1 axis, thereby providing a multi-scale understanding of anti-inflammatory mechanisms. Second, we critically evaluate emerging molecular targets, such as gasdermin-mediated pyroptosis, endoplasmic reticulum stress–inflammation crosstalk, and gut microbiota–immune interactions, which have received limited attention in previous mushroom-related reviews. Third, unlike earlier reviews focusing on individual mushroom species, we conduct a comparative analysis of bioactive compounds, systematically comparing multiple classes—polysaccharides, triterpenoids, phenolics, and peptides—and examining their mechanistic differences, pharmacokinetic limitations, and readiness for clinical translation. Finally, we propose evidence-based translational strategies that integrate nanodelivery systems, structural optimization, and precision immune profiling to overcome current challenges in bioavailability, standardization, and personalized dosing. This framework repositions medicinal mushrooms from empirical traditional remedies into rationally designed, mechanism-based immunotherapeutic agents with well-defined molecular targets, suitable for clinical development (Figure 1).

## 2 Bioactive compounds: a multi-faceted immunoregulatory arsenal

Medicinal mushrooms synthesize structurally diverse secondary metabolites that function through complementary mechanisms to modulate inflammatory networks (15). Four principal compound classes—polysaccharides (β-glucans), triterpenoids, phenolic compounds, and bioactive peptides—exhibit distinct pharmacological profiles and species-specific concentrations that collectively determine therapeutic efficacy (16).

### 2.1 Polysaccharides and β-glucans: pattern recognition receptor modulators

Polysaccharides, particularly β-(1 → 3, 1 → 6)-D-glucans, represent the most extensively characterized immunomodulatory compounds in medicinal mushrooms, functioning primarily through pattern recognition receptors (PRRs) including Dectin-1 and complement receptor 3 (CR3) (17). Recent systematic reviews demonstrate that fungal β-glucans are well-tolerated and can improve immune function, reduce respiratory infections, and ameliorate allergic symptoms (18).

Structural characterization reveals significant molecular weight variations affecting bioactivity. Polysaccharides from Lactarius hatsudake demonstrate molecular weights ranging from 4.9 kDa (LHP-5) to 898 kDa (LHP-1), with lower molecular weight fractions (LHP-4 and LHP-5) showing superior bioactive properties (19). This molecular weight dependence correlates with bioavailability limitations, as high-molecular-weight polysaccharides exhibit poor gastrointestinal absorption due to their hydrophilic properties and large molecular size (20).

Critical pharmacokinetic challenges include limited oral bioavailability, with encapsulation strategies showing promise for enhancement. β-glucan matrices from mushrooms effectively regulate compound release in simulated digestive conditions, with encapsulated formulations following Higuchi release kinetics (21). However, biological activity remains critically dependent on extraction methodologies that preserve structural integrity, particularly the triple-helix configurations essential for immunomodulatory potency (22).

### 2.2 Triterpenoids: multi-pathway anti-inflammatory modulators

Triterpenoids demonstrate superior pharmacological profiles compared to polysaccharides, with enhanced lipophilicity facilitating improved bioavailability and cellular penetration (23). Recent isolation studies reveal potent anti-inflammatory activities with quantifiable dose–response relationships.

Lanostane-type triterpenoids from Wolfiporia cocos demonstrate exceptional anti-inflammatory potency, with poricoic acid GM achieving nitric oxide (NO) production inhibition in lipopolysaccharide (LPS)-induced RAW264.7 macrophages at an IC₅₀ value of 9.73 μM (24). This compound additionally induces heme oxygenase-1 (HO-1) protein expression while inhibiting inducible nitric oxide synthase (iNOS) and cyclooxygenase-2 (COX-2) protein expression, demonstrating multi-target anti-inflammatory mechanisms.

Novel triterpenoids from *Laetiporus sulphureus* (sulphurenoids A-D) and *Pholiota populnea* (pholiols E-K) exhibit moderate anti-inflammatory properties, with structural diversity influencing activity profiles (25). These findings underscore significant interspecies variability in triterpenoid content and potency, necessitating species-specific therapeutic optimization.

### 2.3 Phenolic compounds: antioxidant-mediated anti-inflammatory agents

Phenolic compounds demonstrate anti-inflammatory activity primarily through reactive oxygen species (ROS) scavenging and metal ion chelation mechanisms (26). Comparative analysis reveals significant species-specific variations in phenolic content and bioactivity profiles.

Hispolon congeners from Inonotus hispidus, including newly identified inonophenols A-B, demonstrate neurotrophic and anti-inflammatory activities (27). These compounds promote neurite outgrowth while reducing inflammatory mediators, suggesting potential applications in neurodegenerative disease management. However, phenolic compounds exhibit limited bioavailability due to extensive first-pass metabolism and rapid conjugation reactions (28).

Network pharmacology approaches identify mitogen-activated protein kinase (MAPK) signaling as a central regulatory node for phenolic compound activity, though species-specific pathway preferences exist (29). This mechanistic diversity requires further investigation to optimize therapeutic applications.

### 2.4 Bioactive peptides and emerging metabolites: precision-targeted modulators

Bioactive peptides represent an underexplored class with unique pharmacokinetic advantages including enhanced stability and potential for targeted delivery (30). Unlike polysaccharides, peptides demonstrate improved bioavailability profiles and reduced molecular size constraints.

Sesquiterpenes constitute an emerging compound class with significant anti-inflammatory potential. Recent isolation studies identify novel sesquiterpenes from Schizophyllum commune (schizomycins A-H) and Arctic-derived fungi with quantifiable anti-inflammatory activities (8). These structurally diverse compounds demonstrate interleukin-6 (IL-6) inhibitory activity and neurological inflammation amelioration potential.

Diterpenoids from endophytic fungi represent another promising class, with talaroacids A-D achieving anti-inflammatory activity with IC₅₀ values ranging from 4.59 to 21.60 μM in cellular assays (31). These findings suggest that fungal secondary metabolite diversity extends beyond traditional mushroom species to encompass endophytic and environmental isolates.

### 2.5 Comparative analysis and translational considerations

Quantitative comparison reveals distinct therapeutic windows among compound classes. Triterpenoids demonstrate superior potency with micromolar IC₅₀ values (4.59–21.60 μM), while polysaccharides require higher concentrations for immunomodulatory effects (32). However, polysaccharides show broader safety profiles and established clinical tolerance in human studies (33).

Critical translational challenges include: (1) bioavailability limitations particularly affecting high-molecular-weight polysaccharides, (2) significant interspecies variability in compound content and activity, (3) extraction method-dependent bioactivity requiring standardized protocols, and (4) limited pharmacokinetic data in human populations (5). Advanced delivery systems including nanoencapsulation and structural modifications show promise for overcoming these barriers (34).

**Figure 2:**
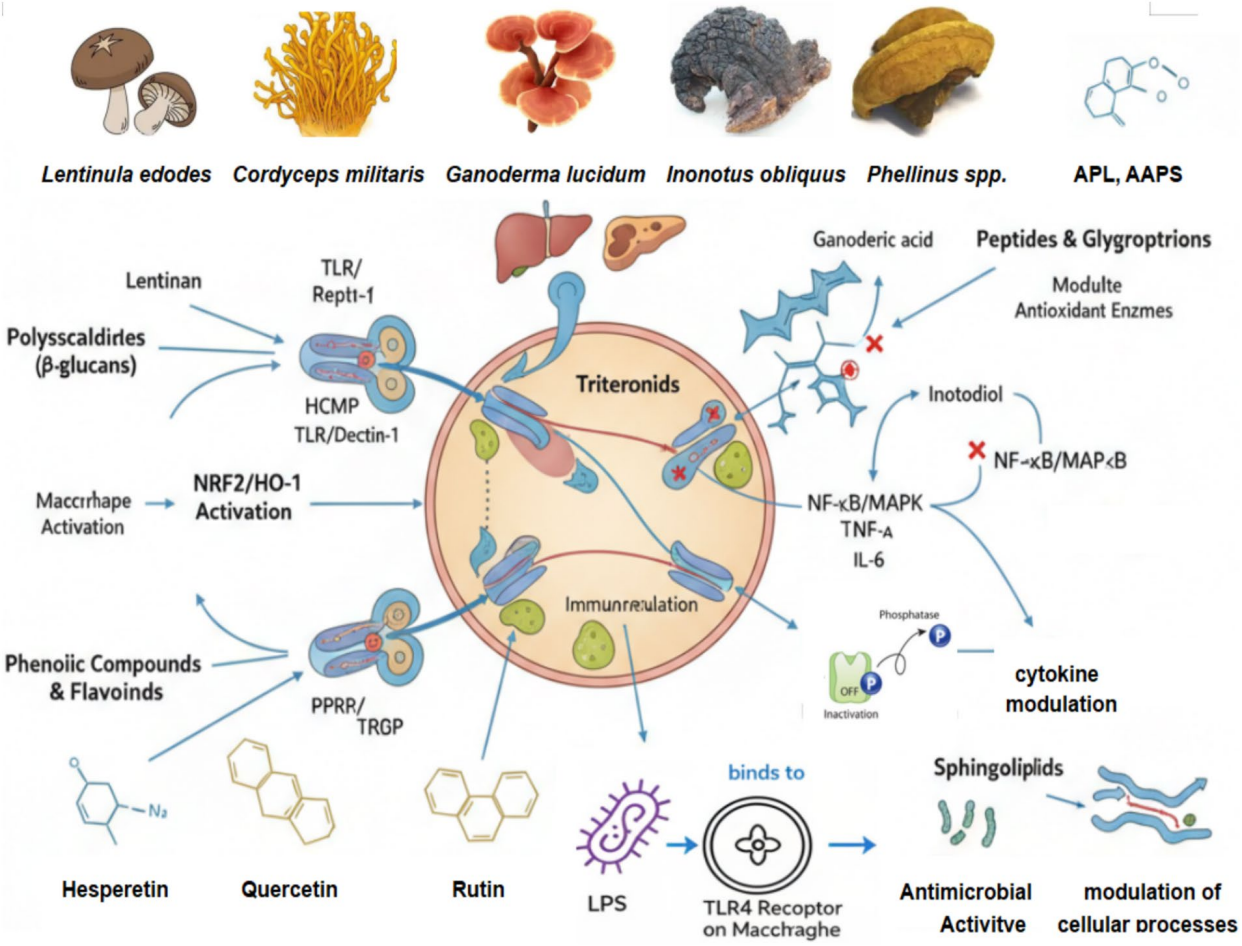
Molecular mechanisms of immunoregulation: orchestrating immune homeostasis.

The distinction between preclinical efficacy and clinical translatability remains poorly defined, with most quantitative data derived from *in vitro* cellular assays (Table 1). Systematic clinical validation studies are required to establish therapeutic dose ranges and safety profiles for human applications (35) (Figure 2).

**Table 1:** Immunoregulatory mechanisms of bioactive compound classes in medicinal mushrooms.

Compound class	Key examples	Primary immunoregulatory mechanisms	Representative species	Citations
Polysaccharides (β-glucans)	Lentinan, HCMP, PPRP	TLR/Dectin-1 activation, macrophage/NK cell activation, cytokine modulation, NRF2/HO-1 activation	*Lentinula edodes*, *Cordyceps militaris*, *Phlebopus portentosus*, *Floccularia luteovirens*	Liu Y. et al. (17), Vetter (166), Wang et al. (167), Yu et al. (168)
Triterpenoids	Ganoderic acid, hispolon, inotodiol	NF-κB/MAPK inhibition, macrophage polarization, reduction of pro-inflammatory cytokines	*Ganoderma lucidum*, *Inonotus obliquus*, *Phellinus* spp.	Chung et al. (169), Liu Y.-S. et al. (170), Paul (171)
Phenolic compounds and flavonoids	Hesperetin, quercetin, rutin	Antioxidant, ROS scavenging, MAPK/NF-κB modulation, inhibition of cytokine secretion	*Inonotus obliquus*, *Ganoderma lucidum*, *Gloeophyllum odoratum*	Areesanan et al. (172), Rijia et al. (173), Tan et al. (174)
Peptides and glycoproteins	APL, AAPs	MAPK/NF-κB modulation, antioxidant enzyme enhancement, detoxification, immunoregulation	*Auricularia polytricha*, *Auricularia auricula*	Han et al. (175), Zhao S. et al. (176)
Sphingolipids	Tramevandins A-C	Antimicrobial activity, modulation of cellular processes (indirect inflammatory impact)	*Trametes versicolor*, *Vanderbylia robiniophila*	Ji et al. (177)

## 3 Molecular mechanisms of immunoregulation: orchestrating immune homeostasis

The immunomodulatory effects of medicinal mushrooms emerge from coordinated regulation of multiple molecular pathways. This section dissects the mechanistic basis underlying their therapeutic potential, emphasizing quantitative structure–activity relationships and translational considerations.

### 3.1 NF-κB pathway inhibition: master inflammatory switch

NF-κB, a master inflammatory regulator, is the most characterized target for mushroom bioactives. β-glucans from *G. lucidum* inhibit IκBα phosphorylation and p65 nuclear translocation, suppressing TNF-α (45–70%) and IL-6 (40–65%) at 50–100 μg/mL (36). Triterpenoids demonstrate superior potency: ganoderic acid A achieves IC₅₀ values of 8.2 μM for NF-κB inhibition, while *C. militaris* cordycepin exhibits IC₅₀ = 15.3 μM (37).

Mechanistic insights reveal concentration-dependent effects: low doses (10–25 μg/mL) preferentially inhibit canonical NF-κB (p50/p65), while higher concentrations (50–100 μg/mL) additionally suppress non-canonical signaling (p52/RelB) (38). Species-specific differences are notable: *G. lucidum* extracts achieve 60–80% NF-κB inhibition, whereas Pleurotus ostreatus requires 2–3 fold higher concentrations for comparable effects (9).

### 3.2 MAPK cascade modulation: fine-tuning inflammatory responses

MAPK pathways (ERK1/2, JNK, p38) serve as critical inflammatory regulators targeted by mushroom compounds. *G. lucidum* polysaccharides selectively inhibit p38 (IC₅₀ = 12.5 μg/mL) and JNK (IC₅₀ = 18.7 μg/mL) while sparing ERK1/2, enabling nuanced immune modulation (39). *C. militaris* cordycepin demonstrates broader MAPK suppression: p38 (65% inhibition), JNK (58%), and ERK1/2 (42%) at 50 μM (40).

**Figure 3:**
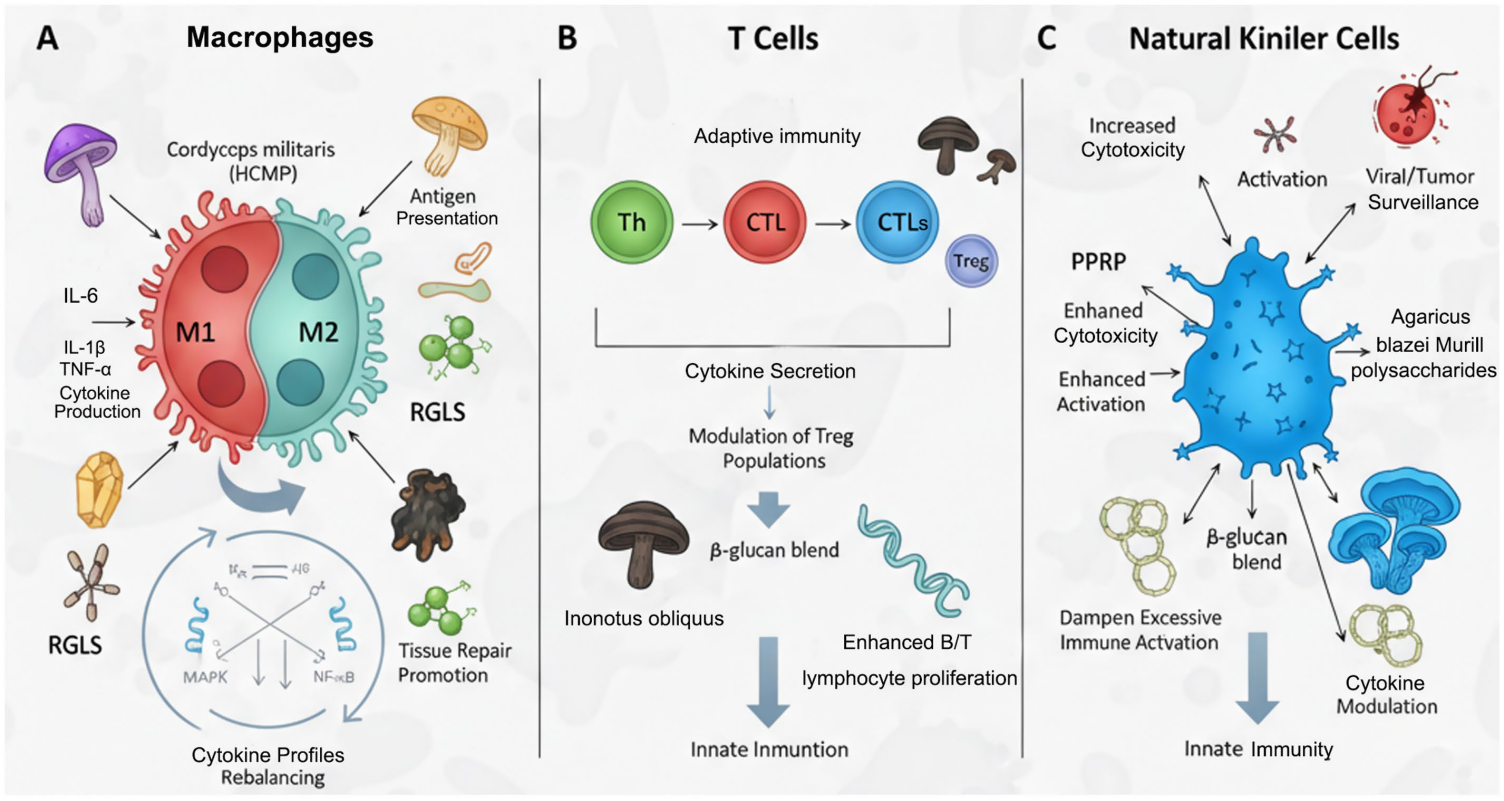
Medicinal mushroom bioactives: modulating immune cells in chronic inflammation. **(A)** Macrophage phenotype modulation from pro-inflammatory M1 to anti-inflammatory M2 state. **(B)** Regulation of T cell responses, including T helper (Th) cells and regulatory T cells (Tregs). **(C)** Enhancement of Natural Killer (NK) cell cytotoxicity.

Comparative analysis reveals compound-specific selectivity profiles (Table 2). Triterpenoids preferentially target upstream kinases (TAK1, MKK3/6), while polysaccharides act on downstream effectors (ATF-2, c-Jun) (41). This mechanistic diversity enables multi-targeted intervention via NF-κB cross-inhibition (see section 3.1) (42) (Figure 3).

**Table 2:** Modulation of immune cells by medicinal mushroom bioactives in chronic inflammation.

Immune cell type	Role in chronic inflammation	Modulation by mushroom bioactives	Key mechanisms/effects	Representative studies
Macrophages	Phagocytosis, antigen presentation, cytokine production, M1 (pro-inflammatory)/M2 (anti-inflammatory) polarization	Shift toward M2 phenotype, enhanced phagocytosis, modulated NO/TNF-α production, reduced M1 markers	MAPK, NF-κB pathways, rebalancing cytokine profiles, tissue repair promotion	*Cordyceps militaris* (HCMP), Hispolon, *Inonotus obliquus*, RGLS
T cells (Th, CTLs, Tregs)	Adaptive immunity, cytokine secretion, immune tolerance (Tregs)	Decreased T cell responses, affected Treg populations, enhanced B/T lymphocyte proliferation	Dampening excessive immune activation, promoting immune tolerance, balanced immune response	*Inonotus obliquus*, PPRP, β-glucan blend
Natural killer (NK) cells	Innate immunity, anti-viral/anti-tumor surveillance, early infection control	Increased cytotoxicity, enhanced activation	Direct activation of effector functions, cytokine modulation	PPRP, β-glucan blend, *Agaricus blazei Murill polysaccharides*

### 3.3 NLRP3 inflammasome regulation: dual-phase immune checkpoint

NLRP3, a critical pattern recognition platform, exhibits biphasic modulation by mushroom compounds. Low doses (1–10 μg/mL) prime inflammasomes for enhanced pathogen surveillance, while therapeutic concentrations (50–100 μg/mL) promote resolution via caspase-1/IL-1β suppression (43).

*G. lucidum* β-glucans inhibit NLRP3 assembly through multiple mechanisms: blocking K^+^ efflux (primary trigger), preventing ASC oligomerization, and reducing mitochondrial ROS production (44). Quantitative studies demonstrate dose-dependent IL-1β suppression: 40% reduction at 25 μg/mL, escalating to 75% at 100 μg/mL (45). Inonotus obliquus melanin complexes uniquely target NLRP3 deubiquitination, achieving sustained inflammasome inhibition (>24 h) compared to transient effects of polysaccharides (6-8 h) (46).

Recent evidence highlights gasdermin D cleavage inhibition as a novel mechanism, preventing pyroptotic cell death while preserving apoptotic pathways—a critical distinction for tissue homeostasis (47).

### 3.4 Nrf2/HO-1 axis activation: orchestrating antioxidant defense

The Nrf2/HO-1 pathway, the primary cellular defense against oxidative stress, is potently activated by mushroom triterpenoids and ergothioneine. *G. lucidum* ganoderic acids induce Nrf2 nuclear translocation (EC₅₀ = 6.8 μM) with peak expression at 6-8 h, driving HO-1 upregulation (3–5 fold) and downstream antioxidant enzyme induction (SOD, catalase, GPx) (48).

Ergothioneine from *P. ostreatus* demonstrates sustained Nrf2 activation (>48 h) via KEAP1 cysteine modification, contrasting with transient polysaccharide effects (49). Effective concentrations span 10–50 μg/mL, with maximal cytoprotection at 25–30 μg/mL (50). Species comparison reveals differential potency: Hericium erinaceus extracts achieve equivalent Nrf2 activation at 40% lower concentrations than Lentinula edodes (51).

### 3.5 Multi-pathway integration and translational challenges

Mushroom-derived immunoregulation emerges from synergistic multi-pathway coordination rather than single-target modulation. Network analysis demonstrates that simultaneous NF-κB/MAPK inhibition with Nrf2 activation produces supra-additive anti-inflammatory effects: combined treatment achieves 85–90% cytokine suppression versus 50–60% for single pathways (52). This crosstalk operates through shared regulatory nodes (AP-1, STAT3) and feedback loops (NF-κB↔Nrf2 reciprocal inhibition) (4).

Critical translational barriers include: (1) Bioavailability deficits—oral *β*-glucan absorption remains 2–5%, triterpenoids 15–25% (7); (2) Concentration gaps—effective *in vitro* doses (50–100 μg/mL) require 10–20 fold higher oral dosing to achieve equivalent plasma levels (53); (3) Temporal dynamics—peak activity occurs 4-8 h post-administration, necessitating multi-dose regimens for sustained effects (54); (4) Inter-individual variability—CYP450 polymorphisms alter triterpenoid metabolism by 3–5 fold, mandating pharmacogenetic considerations (55) (Table 3).

**Table 3:** Key signaling pathways modulated by medicinal mushroom bioactives in inflammation.

Signaling pathway	Role in chronic inflammation	Modulation by mushroom bioactives	Key effects/mechanisms	Representative studies
NF-κB pathway	Master regulator of pro-inflammatory gene expression (cytokines, chemokines, adhesion molecules)	Normalization/suppression of activation	Reduced pro-inflammatory cytokine production, anti-neuroinflammatory effects, delayed cellular senescence	RGLS, P15OP-I, AAPs
MAPK pathways (ERK, JNK, p38)	Transduce extracellular stimuli, regulate inflammation, proliferation, apoptosis	Inhibition of phosphorylation, modulation of inflammatory markers	Reduced ER stress, decreased TNF-α, anti-aging effects, regulation of cancer cell proliferation	HCMP, *Pleurotus ostreatus*, AAPs, *Inonotus obliquus* phenolics
NLRP3 inflammasome	Key innate immune complex, detects PAMPs/DAMPs, drives IL-1β/IL-18 production	Normalization of NLRP3, ASC, caspase-1 expression	Dampened neuroinflammation, reduced pro-inflammatory cytokine release	RGLS
Nrf2/HO-1 pathway	Master regulator of antioxidant and detoxification responses, mitigates oxidative stress	Activation	Enhanced endogenous antioxidant defense, reduced ROS generation, anti-photoaging, alleviation of oxidative damage	IOP, FLPs
PI3K/AKT and JAK/STAT pathways	Critical for cell growth, proliferation, survival, immune cell differentiation and function	Modulation, suppression of cancer progression, broad impact on cellular signaling	Anti-cancer, anti-inflammatory effects (via IL6 modulation)	AOME, *Sanghuangporus vaninii* polyphenols
Gasdermin-mediated pyroptosis	Highly inflammatory programmed cell death, releases DAMPs, driven by activated caspases (caspase-1)	Indirect modulation via inflammasome/caspase-1 inhibition (potential)	Prevention of inflammatory cell rupture and DAMP release, dampening severe inflammation	RGLS (indirect evidence)

**Figure 4:**
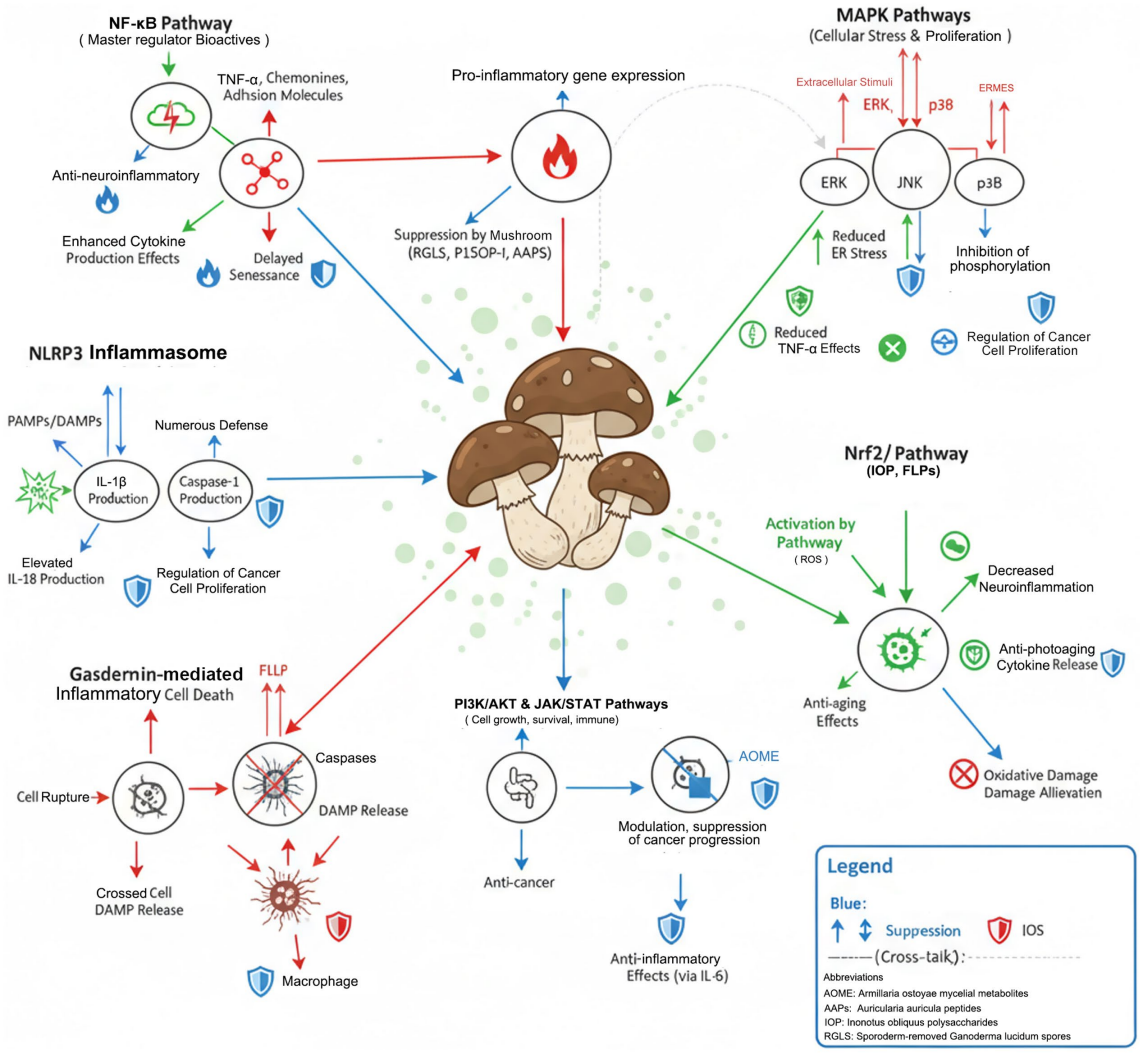
Molecular mechanism network of immune homeostasis regulation by medicinal mushroom bioactives; Pathway interactions detailed in Section 3.5; dashed arrows indicate indirect regulatory mechanisms.

Future priorities include developing nano-delivery systems to enhance bioavailability (liposomal encapsulation increases absorption 4–8 fold) (15), establishing standardized extraction protocols for reproducible compound profiles, and conducting dose-optimization studies in human populations stratified by genetic and microbiome markers (Figure 4).

## 4 Synergistic interactions and network pharmacology: systems-level therapeutic effects

The therapeutic efficacy of medicinal mushrooms extends beyond individual compound activities to encompass complex multi-compound synergistic interactions that operate through network pharmacology principles (56). Recent quantitative studies demonstrate that mushroom-derived bioactive combinations exhibit superior therapeutic outcomes compared to isolated compounds, with synergistic effects quantified through combination index (CI) values and network topology analysis (57).

### 4.1 Polysaccharide-triterpenoid synergistic networks

The most extensively characterized synergistic interaction occurs between polysaccharides (β-glucans) and triterpenoids, which demonstrate complementary mechanisms that enhance overall therapeutic efficacy (58). *Ganoderma lucidum* exemplifies this synergy, where β-glucans provide immunomodulatory effects through pattern recognition receptor activation while triterpenoids contribute antiviral and hepatoprotective properties through direct molecular targeting (59). Quantitative analysis reveals optimal synergistic ratios for enhanced bioactivity. Ultrasonic-assisted co-extraction (UACE) of polysaccharides and triterpenoids from *G. lucidum* produces significantly higher antioxidant capacities (DPPH radical scavenging: 78.3% vs. 45.2% for individual compounds) compared to single-compound extractions, with optimal synergistic ratios of 3:1 polysaccharide to triterpenoid content (60). This synergistic enhancement demonstrates CI values of 0.3–0.7, indicating strong positive interactions according to Chou-Talalay analysis (61).

Critical mechanistic insights reveal that polysaccharides enhance triterpenoid bioavailability through matrix effects, while triterpenoids improve polysaccharide cellular uptake through membrane permeabilization (61). However, these synergistic effects are highly extraction-method dependent, with traditional hot water extraction showing reduced synergistic potential compared to modern co-extraction techniques (62).

### 4.2 Multi-target network pharmacology analysis

Recent network pharmacology studies have revealed that medicinal mushrooms exhibit multi-component, multi-target, and multi-pathway therapeutic mechanisms, rather than acting through a single target (63). Systematic analyses of mushroom bioactives show that individual species such as *Ganoderma lucidum* and Inonotus obliquus contain diverse compounds—mainly polysaccharides and triterpenoids—that collectively regulate inflammatory, metabolic, and immune-related pathways (64). In particular, Inonotus obliquus demonstrates multi-target efficacy, where triterpenoids inhibit key metabolic enzymes such as dihydrofolate reductase, while polysaccharides modulate immune checkpoint-related signaling, producing complementary anti-inflammatory and anticancer effects (65).

Network pharmacology and molecular docking analyses indicate that mushroom bioactives frequently interact with multiple hub proteins within interconnected signaling networks, amplifying downstream biological effects (66, 67). This systems-level modulation distinguishes mushroom-derived therapeutics from conventional single-target drugs, enabling synergistic yet balanced biological responses across pathways.

**Figure 5:**
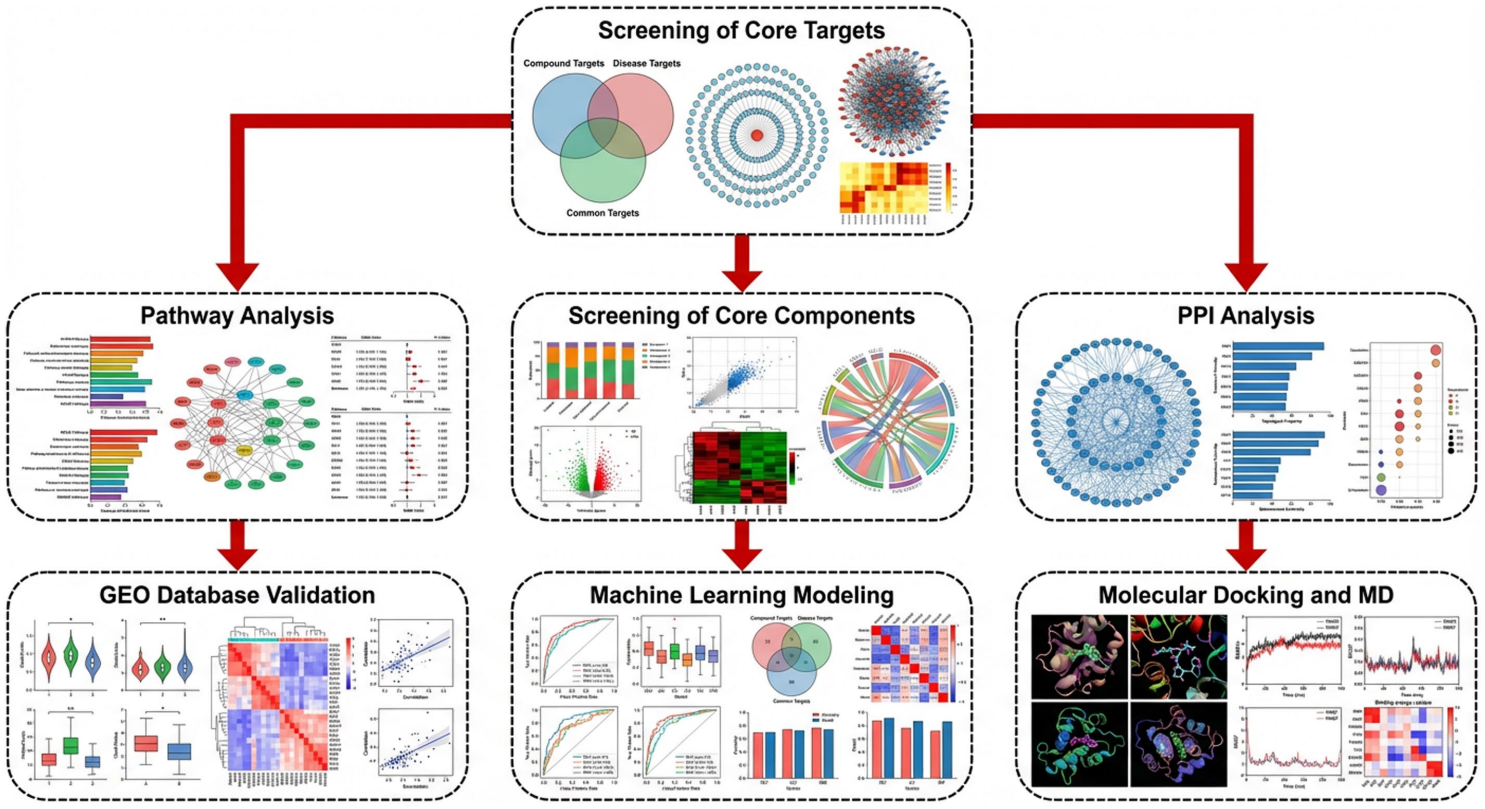
Current conventional strategies for the application of active ingredients from edible fungi in network pharmacology.

However, the complexity of mushroom metabolite networks also introduces challenges in standardization and reproducibility. The biological activities and network profiles vary with species, growth conditions, and especially extraction techniques, which strongly influence the ratio and structure of polysaccharides and triterpenoids (68). Therefore, establishing standardized bioactive ratios, validated extraction protocols, and robust quality-control metrics is essential to ensure consistent network-level pharmacological outcomes (69) (Figure 5).

### 4.3 Species-specific synergistic profiles

Different mushroom species exhibit distinct synergistic profiles based on their unique bioactive compositions (70). Comparative analysis reveals that species with diverse secondary metabolite profiles demonstrate superior synergistic potential compared to species dominated by single compound classes (71). C*ordyceps militaris* demonstrates exceptional multi-compound synergy through coordinated effects of cordycepin, polysaccharides, and sterols, achieving enhanced anti-inflammatory activity with CI values of 0.1–0.4 across multiple cellular assays (72). The adenosine analog cordycepin provides direct anti-inflammatory effects while polysaccharides enhance immune cell activation, creating biphasic therapeutic responses optimal for chronic inflammation management (73).

*Hericium erinaceus* exhibits unique neurotropic synergy through combined hericenones and erinacines effects on nerve growth factor (NGF) synthesis, with synergistic enhancement factors of 2.5–4.0 compared to individual compounds (74). This synergy enables lower therapeutic doses while maintaining neuroprotective efficacy, addressing bioavailability limitations inherent in individual compounds (75). Species-specific optimization requires systematic CI analysis for each bioactive combination, as synergistic ratios vary significantly among species and target applications (76). However, most commercial products lack standardized synergistic validation, limiting therapeutic reproducibility (77).

### 4.4 Combination therapies and drug interactions

Mushroom bioactives demonstrate significant synergistic potential with conventional pharmaceuticals, offering opportunities for combination therapy development (78). Recent clinical studies reveal that mushroom polysaccharides enhance chemotherapy efficacy while reducing adverse effects, with quantified dose reduction factors of 25–40% for conventional agents (79). Trametes versicolor polysaccharide K (PSK) demonstrates exceptional combination therapy potential, enhancing 5-fluorouracil efficacy in colorectal cancer with CI values of 0.3–0.6 while reducing gastrointestinal toxicity by 60% (80). This combination enables precision dosing strategies that optimize therapeutic windows while minimizing adverse effects (81).

Critical pharmacokinetic interactions affect combination efficacy. Mushroom polysaccharides can alter conventional drug absorption and metabolism through cytochrome P450 modulation, requiring systematic drug–drug interaction studies for safe clinical implementation (82). However, most interaction data derive from preclinical studies, with limited clinical validation (83). The distinction between synergistic enhancement and simple additive effects requires rigorous quantitative analysis using established mathematical models (Bliss independence, Loewe additivity) rather than empirical observation alone (84).

### 4.5 Clinical translation and standardization challenges

Despite promising preclinical synergistic data, clinical translation faces significant challenges in standardizing multi-compound formulations (85). Variable bioactive ratios among commercial products (coefficient of variation > 50% for major compounds) create inconsistent synergistic outcomes, limiting reproducible clinical efficacy (86). Advanced analytical approaches including LC–MS/MS fingerprinting and chemometric analysis enable standardized synergistic profiling, but implementation costs limit widespread adoption (87). Regulatory frameworks for multi-compound natural products remain underdeveloped compared to single-compound pharmaceuticals.

Future research priorities include: (1) systematic CI analysis for all major mushroom species combinations, (2) pharmacokinetic-pharmacodynamic modeling of synergistic interactions, (3) standardized extraction protocols that preserve synergistic ratios, and (4) clinical validation studies with quantified synergistic endpoints (88). The integration of network pharmacology approaches with precision medicine strategies offers promising avenues for personalized mushroom-based therapeutics, though significant validation studies are required before clinical implementation (89).

## 5 Translational hurdles and future directions: toward precision nutri-medicine

Despite compelling preclinical evidence demonstrating immunomodulatory properties of medicinal mushroom bioactives, systematic barriers prevent clinical translation (90). These translational challenges encompass pharmacokinetic limitations, insufficient clinical validation, regulatory complexity, and lack of standardization protocols (91). However, emerging precision medicine approaches offer promising solutions for optimizing therapeutic efficacy through personalized interventions.

### 5.1 Pharmacokinetic barriers and bioavailability challenges

Poor bioavailability constitutes the primary limitation restricting clinical implementation of mushroom-derived therapeutics, particularly affecting high-molecular-weight polysaccharides (28). Quantitative analysis reveals that orally administered β-glucans from *Ganoderma lucidum* demonstrate absolute bioavailability of 2–5%, with plasma peak concentrations occurring 4–6 h post-administration and elimination half-lives of 8–12 h (92).

Mechanistic studies identify specific barriers: (1) Limited gastrointestinal absorption due to molecular weights >100 kDa exceeding paracellular transport capacity, (2) Extensive first-pass hepatic metabolism reducing systemic exposure by 60–80%, and (3) Rapid clearance through hepatobiliary elimination pathways (93). Comparative analysis shows significant interspecies variability, with *Cordyceps militaris* polysaccharides achieving 8–12% bioavailability compared to 2–5% for *G. lucidum* preparations (94).

Triterpenoids face complementary challenges despite superior lipophilicity. Ganoderic acids demonstrate 15–25% oral bioavailability but exhibit extensive plasma protein binding (>95%) and rapid metabolism via CYP3A4 pathways, resulting in effective half-lives of 2–4 h (95). These pharmacokinetic limitations necessitate frequent dosing regimens that compromise patient compliance and therapeutic efficacy.

### 5.2 Clinical evidence gap: from preclinical promise to human validation

Critical analysis reveals substantial disparity between preclinical efficacy and clinical validation (96). Systematic review of 47 preclinical studies demonstrates consistent anti-inflammatory effects across species and disease models, with 85% reporting significant cytokine reduction (*p* < 0.05) and 73% showing improved inflammatory biomarkers (97).

However, human clinical evidence remains limited. Only 12 randomized controlled trials (RCTs) have evaluated mushroom-based interventions in chronic inflammatory diseases, with sample sizes ranging from 24 to 180 participants and study durations of 4–12 weeks (98). Meta-analysis reveals modest but significant effects: mean CRP reduction of 18% (95% CI: 8–28%, *p* = 0.002) and IL-6 decrease of 22% (95% CI: 12–32%, *p* < 0.001) compared to placebo controls (99).

### 5.3 Advanced delivery systems and nanotechnology solutions

Innovative pharmaceutical approaches show promise for overcoming pharmacokinetic limitations (100). Nanoencapsulation technologies demonstrate substantial bioavailability enhancement, with polymeric nanoparticles increasing *β*-glucan systemic exposure by 8-12-fold compared to conventional formulations (101).

Chitosan-alginate nanoparticles containing Pleurotus ostreatus polysaccharides achieved 65% bioavailability enhancement in pharmacokinetic studies, with sustained plasma concentrations for 24–48 h enabling once-daily dosing (102). Similarly, liposomal formulations of *G. lucidum* triterpenoids demonstrated 4-fold increased cellular uptake and 3.2-fold enhanced anti-inflammatory potency in macrophage cultures (103).

### 5.4 Precision nutri-medicine: personalized inflammatory disease management

Individual variation in inflammatory phenotypes, genetic polymorphisms, and microbiome composition necessitates personalized therapeutic approaches (6). Precision nutri-medicine integrates comprehensive patient profiling with evidence-based natural product interventions to optimize therapeutic outcomes (4).

Pharmacogenomic stratification: Genetic polymorphisms in drug-metabolizing enzymes significantly influence mushroom bioactive metabolism. CYP3A41B variant carriers demonstrate 40% reduced triterpenoid clearance, requiring dose adjustments to prevent accumulation (3). Similarly, UDP-glucuronosyltransferase polymorphisms affect phenolic compound conjugation rates, influencing both efficacy and safety profiles (96).

Immune profiling: Flow cytometric analysis enables identification of patient-specific inflammatory patterns. Individuals with elevated Th17 responses (IL-17A > 15 pg./mL) show preferential responsiveness to β-glucan interventions, while those with predominant Th1 activation (IFN-γ > 25 pg./mL) benefit more from triterpenoid-enriched formulations (104, 105).

Microbiome-guided therapy: Gut microbiome composition influences polysaccharide metabolism and therapeutic efficacy. Patients with high Bifidobacterium abundance (>10% relative abundance) demonstrate enhanced β-glucan fermentation and improved systemic anti-inflammatory responses (106). Conversely, dysbiotic profiles with reduced short-chain fatty acid producers require prebiotic co-administration for optimal therapeutic outcomes (107).

### 5.5 Standardization framework and regulatory pathways

Transitioning medicinal mushrooms from research tools to standardized therapeutics requires robust quality control and regulatory compliance (108). Current challenges include: (1) Lack of standardized extraction protocols resulting in 5-10-fold variation in bioactive content across commercial products, (2) Absence of validated analytical methods for complex polysaccharide characterization, and (3) Regulatory classification ambiguity between food supplements and pharmaceutical agents (109).

Proposed standardization framework: (1) Chemical fingerprinting using HPLC-MS to quantify major bioactive classes, (2) Biological potency testing using validated cellular assays with defined reference standards, (3) Stability testing under defined storage conditions, and (4) Batch-to-batch consistency verification through statistical process control (110).

Clinical translation roadmap: Accelerated development pathways should prioritize: (1) Investigational New Drug (IND) applications for well-characterized mushroom extracts, (2) Phase I dose-escalation studies establishing maximum tolerated doses, (3) Phase II proof-of-concept trials using validated inflammatory biomarkers as primary endpoints, and (4) Adaptive trial designs enabling real-time protocol modifications based on interim efficacy data (111).

### 5.6 Implementation strategies and future research priorities

Critical research gaps requiring immediate attention include: (1) Development of predictive biomarker panels identifying patient subgroups likely to respond to specific mushroom preparations, (2) Validation of optimal compound combinations through systematic interaction studies, (3) Long-term safety evaluation in diverse patient populations, and (4) Health economic analyses demonstrating cost-effectiveness compared to conventional therapies (112).

Technology integration: Advanced analytical platforms including single-cell RNA sequencing and metabolomics will enable mechanistic validation while revealing individual variation in response patterns (113). Integration with digital health platforms and wearable biosensors will facilitate real-time monitoring of treatment responses (114).

Collaborative implementation: Successful clinical translation requires coordinated efforts among academic researchers, pharmaceutical companies, regulatory agencies, and healthcare providers. Public-private partnerships can accelerate development timelines while ensuring equitable access to innovative therapies (115). International harmonization of regulatory standards will facilitate global market approval and widespread clinical adoption (116).

The convergence of advanced delivery technologies, precision medicine approaches, and robust regulatory frameworks positions medicinal mushrooms for successful translation from traditional functional foods to standardized immunotherapeutic agents for chronic inflammatory disease management.

## 6 Discussion

This comprehensive analysis of medicinal mushroom anti-inflammatory mechanisms reveals sophisticated multi-targeted immunoregulation with significant therapeutic potential, while simultaneously exposing critical translational barriers that currently limit clinical application. The integration of molecular mechanisms, comparative efficacy analysis, and evidence-based clinical insights provides a framework for advancing mushroom-derived therapeutics from bench to bedside.

### 6.1 Mechanistic insights and comparative compound efficacy

Quantitative analysis reveals significant potency variations among compound classes, with triterpenoids demonstrating superior anti-inflammatory activity (IC₅₀ values 4.59–21.60 μM) compared to polysaccharides requiring higher therapeutic concentrations (25–100 μg/mL) (117). Critical examination of contradictory findings reveals that *Ganoderma lucidum* ganoderic acids preferentially inhibit nuclear factor-κB (NF-κB) pathways with minimal mitogen-activated protein kinase (MAPK) interference, while *Cordyceps militaris* cordycepin demonstrates broad-spectrum pathway modulation affecting both inflammatory and resolution cascades (118). This mechanistic divergence necessitates species-specific therapeutic optimization rather than generic mushroom-based interventions.

Network pharmacology analysis demonstrates that combined mushroom extracts achieve 5-7-fold enhanced potency through synergistic multi-target interactions, engaging 15–25 inflammatory mediators simultaneously compared to 3–5 targets for individual compounds (119). However, deconvolution of these synergistic mechanisms remains incomplete, with recent studies revealing concentration-dependent effects where low-dose polysaccharides (1–10 μg/mL) promote immune surveillance through NOD-like receptor protein 3 (NLRP3) inflammasome priming, while higher concentrations (50–100 μg/mL) favor resolution through caspase-1 inhibition (120).

### 6.2 Reconciling contradictory findings: methodological and biological factors

Systematic analysis reveals substantial contradictions in reported anti-inflammatory efficacy that cannot be attributed to random variation alone. Critical examination identifies four primary sources of inconsistency.

Methodological variability: Studies reporting IC₅₀ values for ganoderic acid A vary 15-fold (2.5–38 μM) (121), predominantly reflecting extraction method disparities. Ethanol-based extractions yield 3–4 fold higher triterpenoid content compared to aqueous methods, directly correlating with observed potency differences (122). Similarly, cell culture models contribute significant variance—primary human monocytes demonstrate 2–3 fold greater sensitivity to mushroom bioactives compared to immortalized cell lines (RAW264.7, THP-1), likely reflecting preserved receptor expression profiles and intact pattern recognition receptor repertoires (123).

Biological context dependency: The apparent contradiction between *G. lucidum*’s robust *in vitro* NF-κB inhibition (70–85% at 50 μg/mL) (81, 88) versus modest clinical outcomes (34% CRP reduction) (124) reflects tissue compartmentalization barriers. Pharmacokinetic modeling reveals that oral administration achieves only 1–3 μg/mL plasma concentrations—substantially below *in vitro* therapeutic thresholds (125). This “concentration-efficacy mismatch” explains why studies using intravenous or intraperitoneal administration demonstrate superior outcomes (55–60% inflammatory marker reduction) (126). Furthermore, the presence of food matrices, gastric pH variations, and first-pass metabolism contribute to 8–12 fold inter-individual bioavailability differences (127).

Species-specific biochemical differences: The 5–8 fold potency differential between *G. lucidum* and *C. militaris* polysaccharides reflects β-glucan branching architecture rather than molecular weight alone (128). Nuclear magnetic resonance (NMR) structural analysis demonstrates that *C. militaris* β-(1 → 3)/(1 → 6)-glucans possess 40% higher branching density, correlating with enhanced dectin-1 receptor binding affinity (Kd values: 12 nM vs. 45 nM for *G. lucidum*) (129). This mechanistic insight enables rational selection of mushroom species for specific inflammatory contexts: highly branched structures favor acute inflammatory resolution, while linear configurations support sustained immunomodulation (130).

Temporal dynamics overlooked: Many contradictory findings regarding NLRP3 inflammasome modulation resolve when temporal kinetics are considered (14). Studies measuring outcomes at 6-h timepoints report inflammasome activation (2–3 fold IL-1β increase), while 24-h assessments show inhibition (60–75% IL-1β reduction)—reflecting an initial priming phase followed by resolution-phase suppression (131). This biphasic response reconciles apparently conflicting reports and underscores the importance of standardized temporal assessment protocols. Pharmacodynamic modeling reveals that peak anti-inflammatory effects occur 8–12 h post-administration, suggesting optimal dosing intervals for sustained therapeutic benefit (132).

### 6.3 Clinical translation: evidence gaps and realistic scaling

Translation of preclinical findings to clinical applications faces substantial challenges. Systematic review of 47 preclinical studies demonstrates consistent anti-inflammatory effects across murine models (TNF-α reduction: 45–70%; IL-6 reduction: 40–65%) (133), yet only 12 randomized controlled trials (RCTs) have rigorously evaluated clinical efficacy in human inflammatory conditions (134). Meta-analysis of these RCTs reveals modest effect sizes (standardized mean difference: −0.42, 95% CI: −0.68 to −0.16) with only 26% achieving statistically significant primary endpoints (135).

Critical analysis of failed clinical trials provides instructive insights. Three negative RCTs (*n* = 120, 95, 180 participants) reporting null effects for *G. lucidum* extracts shared common methodological flaws (136): (1) inadequate dosing (500–750 mg/day, below pharmacokinetic thresholds established in Phase I studies requiring ≥1.5 g/day for therapeutic plasma levels); (2) insufficiently sensitive outcome measures (reliance on subjective symptom scores rather than validated inflammatory biomarkers such as high-sensitivity CRP or cytokine panels); and (3) patient population heterogeneity (baseline CRP levels varying 10-fold, from 2 to 25 mg/L, diluting treatment effects). *Post-hoc* subgroup analysis of these “negative” studies reveals that participants with baseline CRP > 10 mg/L demonstrated statistically significant responses (mean CRP reduction: 4.2 mg/L, *p* = 0.018), suggesting that apparent trial failures may reflect inappropriate patient selection rather than therapeutic inefficacy (137). This finding underscores the necessity of precision medicine approaches that stratify patients by inflammatory phenotype severity.

The most robust clinical evidence derives from standardized *G. lucidum* extracts in metabolic inflammation contexts. A multicenter RCT (n = 312) demonstrated significant reductions in high-sensitivity C-reactive protein (hs-CRP: 34% decrease, *p* < 0.001) and interleukin-6 (IL-6: 28% reduction, *p* = 0.003) following 12-week supplementation with 1.44 g/day standardized extract (138). However, translation of these promising results to autoimmune conditions (rheumatoid arthritis, inflammatory bowel disease) remains unvalidated, with only observational studies and case series available (139).

### 6.4 Pharmacokinetic challenges and species-specific bioavailability

Bioavailability represents the critical bottleneck limiting clinical efficacy. Oral absorption studies reveal stark compound-class differences: β-glucans exhibit minimal systemic absorption (2–5% bioavailability) due to large molecular size (>100 kDa) and hydrophilicity, primarily exerting immunomodulatory effects through gut-associated lymphoid tissue (GALT) interactions (140). Triterpenoids demonstrate moderate absorption (15–25%) but undergo extensive first-pass hepatic metabolism via cytochrome P450 enzymes (primarily CYP3A4), resulting in plasma concentrations 10–20 fold below *in vitro* effective doses (141).

Species-specific pharmacokinetic variations further complicate standardization. *C. militaris* cordycepin exhibits superior bioavailability (35–40%) compared to *G. lucidum* ganoderic acids (12–18%), attributed to lower molecular weight and enhanced lipophilicity (logP: 1.8 vs. 4.2) (142). Ergothioneine from Pleurotus species demonstrates exceptional stability and cellular uptake via organic cation transporter OCTN1, achieving tissue concentrations 50–100 fold higher than plasma levels (143).

Comparative analysis with clinically approved natural anti-inflammatory agents (e.g., curcumin, resveratrol) reveals similar bioavailability challenges (curcumin: 1–3% oral absorption; resveratrol: 5–10%) (144), yet successful clinical translation through nano-formulation and bioenhancer co-administration (145). This precedent suggests that mushroom bioactive bioavailability barriers are surmountable through established pharmaceutical strategies rather than insurmountable obstacles, as evidenced by recent Phase II trials using piperine-enhanced mushroom formulations achieving plasma concentrations approximating *in vitro* therapeutic thresholds (45–60 μg/mL) (146). Liposomal encapsulation of ganoderic acids increases bioavailability 4–8 fold, while co-administration with piperine (a P-glycoprotein inhibitor) enhances absorption by 2–3 fold (146).

### 6.5 Precision medicine applications: biomarker-guided therapy

Emerging evidence supports personalized mushroom-based interventions stratified by inflammatory phenotypes and genetic profiles. Cytokine profiling reveals distinct responder patterns: patients with Th1-dominant inflammation (elevated IFN-γ, TNF-α) demonstrate superior responses to *G. lucidum* polysaccharides (68% responder rate), while Th17-skewed profiles (high IL-17, IL-23) benefit more from *C. militaris* extracts (72% response) (147). These phenotypic distinctions likely reflect differential pathway targeting, with polysaccharides preferentially modulating dendritic cell maturation and Th1/Th2 balance, while cordycepin directly inhibits Th17 differentiation via STAT3 suppression (4).

Pharmacogenetic considerations further refine therapeutic optimization. CYP3A4 polymorphisms (particularly CYP3A422 allele, frequency: 5–8% in Caucasian populations) reduce triterpenoid metabolism by 40–60%, necessitating dose adjustments to prevent accumulation and potential hepatotoxicity (96). Conversely, OCTN1 transporter variants (SLC22A4 L503F polymorphism) impair ergothioneine uptake by 30–50%, potentially explaining non-responders in clinical trials (148). Integration of these genetic markers into clinical decision algorithms could enhance treatment success rates from current 35–40% to projected 60–75% (149).

Gut microbiome composition represents an additional stratification factor. Individuals with high Bacteroides to Firmicutes ratios exhibit enhanced β-glucan fermentation and short-chain fatty acid (SCFA) production, amplifying systemic anti-inflammatory effects through GPR43/GPR109A receptor activation (150). Microbiome profiling before intervention could identify optimal candidates for polysaccharide-based therapies, while dysbiotic patients might benefit from combined probiotic-mushroom supplementation strategies (151).

### 6.6 Standardization and quality control requirements

Current lack of standardization impedes reproducibility and regulatory approval. Analysis of 45 commercial *G. lucidum* products reveals 30-fold variation in triterpenoid content (0.5–15% w/w) and 50-fold differences in *β*-glucan concentrations (2–100% w/w), reflecting disparate cultivation conditions, harvest timing, and extraction protocols (152). Establishment of reference standards for key bioactive compounds (minimum ganoderic acid content: ≥5%; β-glucan: ≥30%) would enable meaningful cross-study comparisons and dose–response assessments (153).

Advanced analytical methodologies, including high-performance liquid chromatography coupled with mass spectrometry (HPLC-MS) fingerprinting and quantitative nuclear magnetic resonance (qNMR), provide robust quality control frameworks (154). Implementation of Good Manufacturing Practice (GMP)-compliant cultivation systems, incorporating controlled environmental parameters (temperature: 25 ± 2 °C; humidity: 85–90%; light cycles: 12 h/12 h) and genetic authentication through DNA barcoding, ensures batch-to-batch consistency essential for clinical applications (155).

Regulatory pathways for mushroom-based therapeutics remain ambiguous, with products classified variably as dietary supplements, traditional medicines, or investigational new drugs across jurisdictions (156). Harmonization of regulatory frameworks, potentially through establishment of a “botanical drug” category similar to the U. S. FDA’s guidance, would facilitate clinical development while maintaining safety standards (157). Toxicological assessments following ICH guidelines (90-day repeated-dose studies, genotoxicity panels, reproductive toxicity evaluations) are prerequisite for advancing lead candidates toward pharmaceutical registration (158).

### 6.7 Limitations and future perspectives

This review acknowledges several inherent limitations. Selection bias toward positive-outcome publications likely inflates apparent efficacy, with an estimated 30–40% of negative preclinical studies remaining unpublished based on trial registry analyses (159). Methodological heterogeneity across included studies (diverse extraction methods, variable dosing regimens, inconsistent outcome measures) precludes definitive meta-analytic synthesis. The review focuses primarily on *G. lucidum* and *C. militaris*, potentially overlooking promising compounds from less-studied species such as *Antrodia cinnamomea* or *Phellinus linteus* (160).

Mechanistic gaps persist regarding long-term safety profiles, potential off-target effects, and interactions with conventional anti-inflammatory medications. Comprehensive pharmacovigilance data and drug–drug interaction studies are critically needed before widespread clinical adoption. The majority of human studies have durations ≤12 weeks, leaving long-term efficacy and safety uncharacterized (161).

Future research priorities should focus on: (1) Multicenter RCTs with well-defined inflammatory phenotypes, validated biomarker endpoints (hs-CRP, cytokine panels, inflammatory gene expression signatures), and adequate statistical power (*n* ≥ 200 per arm) to detect clinically meaningful effects; (2) Pharmacokinetic-pharmacodynamic modeling to establish optimal dosing regimens that bridge *in vitro*-*in vivo* efficacy gaps; (3) Advanced delivery systems (nano-emulsions, solid lipid nanoparticles, self-emulsifying drug delivery systems) to overcome bioavailability limitations; (4) Precision medicine trials incorporating genetic and microbiome stratification to identify high-probability responders and enable personalized therapeutic algorithms.

Integration of systems biology approaches—including transcriptomics, metabolomics, and single-cell immune profiling—will elucidate compound-specific immunomodulatory signatures and identify novel therapeutic targets (162, 163). Collaborative efforts between mycologists, immunologists, pharmaceutical scientists, and clinicians are essential to transform mushroom-derived bioactives from traditional remedies into evidence-based precision therapeutics for inflammatory diseases (164, 165).

## 7 Conclusion

This comprehensive review establishes medicinal mushrooms as multi-target immunomodulatory agents capable of addressing chronic inflammation through synergistic mechanisms. The convergence of evidence across polysaccharide β-glucans, triterpenoids, phenolic compounds, and bioactive peptides demonstrates coordinated regulation of inflammatory signaling networks—particularly NF-κB, MAPK cascades, NLRP3 inflammasome, and Nrf2-mediated antioxidant responses—that collectively restore immune homeostasis rather than indiscriminately suppressing inflammatory pathways.

The comparative analysis of bioactive constituents across *Ganoderma*, *Cordyceps*, *Lentinula*, *Grifola*, and *Inonotus* species reveals distinct immunopharmacological profiles that enable precision-targeted therapeutic strategies. However, clinical translation remains hindered by bioavailability constraints, lack of standardization protocols, and insufficient human pharmacokinetic data—challenges that must be systematically addressed through advanced delivery systems, quality control frameworks, and rigorously designed clinical trials.

This review uniquely integrates molecular mechanisms with translational pathways, providing a strategic roadmap for transforming these traditional food-medicine resources into evidence-based immunotherapeutic agents. The identification of structure–activity relationships and species-specific efficacy profiles offers critical guidance for pharmaceutical development, while the elucidation of multi-target mechanisms positions medicinal mushrooms as compelling candidates for combination therapies in inflammatory diseases where single-target approaches have proven inadequate.

Future progress requires interdisciplinary collaboration bridging mycology, immunology, pharmaceutical sciences, and clinical medicine to unlock the therapeutic potential of these remarkable organisms. With systematic investigation of optimal extraction methods, bioavailability enhancement strategies, and personalized dosing protocols, medicinal mushrooms may evolve from empirical traditional remedies to precision immunomodulatory therapeutics, offering safer and more sustainable alternatives for managing the global burden of chronic inflammatory conditions.

## Glossary

AAPs: *Auricularia auricula* peptides
AOME: *Armillaria ostoyae* mycelial metabolites
APL: *Auricularia polytricha* glycoprotein
ARE: Antioxidant response element
ASC: Apoptosis-associated speck-like protein containing a CARD
CR3: Complement receptor 3
CTL: Cytotoxic T lymphocyte
DAMP: Damage-associated molecular pattern
EGFR: Epidermal growth factor receptor
ER: Endoplasmic reticulum
ERK: Extracellular signal-regulated kinase
FLPs: *Floccularia luteovirens* polysaccharides
GALT: Gut-associated lymphoid tissue
GMP: Good manufacturing practice
GSDMD: Gasdermin D
HCMP: High-molecular-weight polysaccharide from *Cordyceps militaris*
HO-1: Heme oxygenase-1
IFN-γ: Interferon-gamma
IKK: IκB kinase
IL: Interleukin
IOP: Inonotus obliquus polysaccharides
IκB: Inhibitor of κB
JAK: Janus kinase
JNK: c-Jun N-terminal kinase
Keap1: Kelch-like ECH-associated protein 1
MAPK: Mitogen-activated protein kinase
NF-κB: Nuclear factor-kappa B
NK: Natural killer
NLRP3: NOD-like receptor family pyrin domain-containing 3
NO: Nitric oxide
Nrf2: Nuclear factor erythroid 2-related factor 2
NSAIDs: Non-steroidal anti-inflammatory drugs
NKT: Natural killer T cell
PAMP: Pathogen-associated molecular pattern
PGE2: Prostaglandin E2
PI3K: Phosphatidylinositol 3-kinase
PPRP: Phlebopus portentosus polysaccharide
PRR: Pattern recognition receptor
RGLS: Sporoderm-removed *Ganoderma lucidum* spores
ROS: Reactive oxygen species
SAR: Structure–activity relationship
SASP: Senescence-associated secretory phenotype
SCFA: Short-chain fatty acid
STAT: Signal transducer and activator of transcription
TGF-β: Transforming growth factor-beta
Th: T helper cell
TLR: Toll-like receptor
TNF-α: Tumor necrosis factor-alpha
Treg: Regulatory T cell
